# Whole-genome sequencing of *Alcaligenes* sp. strain MMA: insight into the antibiotic and heavy metal resistant genes

**DOI:** 10.3389/fphar.2023.1144561

**Published:** 2023-05-11

**Authors:** Kushneet Kaur Sodhi, Chandra Kant Singh, Mohit Kumar, Dileep Kumar Singh

**Affiliations:** ^1^ Sri Guru Tegh Bahadur Khalsa College, University of Delhi, Delhi, India; ^2^ Sri Aurobindo College, University of Delhi, Delhi, India; ^3^ Hindu College, University of Delhi, Delhi, India; ^4^ Department of Zoology, University of Delhi, Delhi, India

**Keywords:** *Alcaligenes* sp. MMA, antibiotic-resistant genes, draft genome, metal resistance genes, whole-genome sequencing

## Abstract

**Introduction:** A wide range of pollutants, including the likes of xenobiotics, heavy metals, and antibiotics, are characteristic of marine ecosystems. The ability of the bacteria to flourish under high metal stress favors the selection of antibiotic resistance in aquatic environments. Increased use and misuse of antibiotics in medicine, agriculture, and veterinary have posed a grave concern over antimicrobial resistance. The exposure to these heavy metals and antibiotics in the bacteria drives the evolution of antibiotic and heavy metal resistance genes. In the earlier study by the author *Alcaligenes* sp. MMA was involved in the removal of heavy metals and antibiotics. *Alcaligenes* display diverse bioremediation capabilities but remain unexplored at the level of the genome.

**Methods:** To shed light on its genome, the *Alcaligenes* sp. strain MMA, was sequenced using Illumina Nova Seq sequencer, which resulted in a draft genome of 3.9 Mb. The genome annotation was done using Rapid annotation using subsystem technology (RAST). Given the spread of antimicrobial resistance and the generation of multi-drug resistant pathogens (MDR), the strain MMA was checked for potential antibiotic and heavy metal resistance genes Further, we checked for the presence of biosynthetic gene clusters in the draft genome.

**Results:** *Alcaligenes* sp. strain MMA, was sequenced using Illumina Nova Seq sequencer, which resulted in a draft genome of 3.9 Mb. The RAST analysis revealed the presence of 3685 protein-coding genes, involved in the removal of antibiotics and heavy metals. Multiple metal-resistant genes and genes conferring resistance to tetracycline, beta-lactams, and fluoroquinolones were present in the draft genome. Many types of BGCs were predicted, such as siderophore. The secondary metabolites of fungi and bacteria are a rich source of novel bioactive compounds which have the potential to in new drug candidates.

**Discussion:** The results of this study provide information on the strain MMA genome and are valuable for the researcher in further exploitation of the strain MMA for bioremediation. Moreover, whole-genome sequencing has become a useful tool to monitor the spread of antibiotic resistance, a global threat to healthcare.

## Introduction

The genus *Alcaligenes* is ubiquitous and is present in water and soil. They are also found to be associated with humans. The bacteria are not pathogenic but are regarded as opportunistic pathogens in humans (Cruz et al., 2021). *Alcaligenes* is a Gram-negative bacterium that belongs to the family Alcaligenaceae within the order Burkholderiales. The type species is *Alcaligenes faecalis*. *A.faecalis* consists of three subspecies, namely, *A.faecalis* subsp. *phenolicus, A.faecalis* subsp. *faecalis*, *A.faecalis* subsp. *parafaecalis,* this genus, three other species are described, namely, *A. pakistanensis, A. aquatilis,* and *A. endophyticus* (Mehanni et al., 2019).

Antibiotics and heavy metals are emerging contaminants and are potentially harmful to animals and humans. The potential threat of antibiotics and metals to human health has raised important concerns. Both antibiotic and metal-resistant bacteria are found in most environments. The ability of the bacteria to thrive at high metal stress tends to favor the selection of antibiotic-resistant bacteria in aquatic environments, with a risk of spreading resistance to human pathogens (Bao et al., 2020). Metal contamination in nature has a vital role in the maintenance and emergence of antibiotic-resistant genes along with metal resistance in microorganisms. The increase in the HMs resistance has a serious impact, as it may significantly contribute to the evolution of ARGs which is due to the selective pressure (Squadrone, 2020). Multidrug resistance is now a major environmental safety hazard on a global scale. It has been proven by numerous research that heavy metals can encourage the spread of bacterial resistance. One of the findings by Xu et al., 2022, demonstrated a significant increase in the minimum inhibitory concentration of *E. coli* and *Staphylococcus aureus* on antibiotics, particularly norfloxacin, and tetracyclines, and a higher rate of cross-resistance, indicating that heavy metals can cause bacteria to acquire antibiotic resistances that were not previously present. After heavy metal exposure, it was found that the expression of other efflux pump and resistance genes in *Escherichia coli* was upregulated, particularly tetB, tolC, and arcAB genes. This finding suggests that heavy metals may increase antibiotic resistance by altering the expression of efflux pumps. According to Knapp et al., the abundance of many resistance genes (tetM, tetW, blaOXA, ermB, and ermF) had a substantial positive connection with the quantity of Cu in the soil (Knapp et al., 2011). Similar to this, it has been demonstrated that metal levels of Chromium, Cadmium, Nickel, and Arsenic are substantially linked with the abundance of resistance genes such sul1, sul2, tetM, tetQ, ermB, and mef A in landfill leachate (Gao et al., 2022). Environmental *E. coli* isolates frequently exhibit multiple antibiotic resistance when exposed to metals, such as resistance to beta-lactam antibiotics when exposed to copper and zinc in pig manure (Hölzel et al., 2012) and an increase in resistant isolates of *E. coli* in the gut microbiota of piglets when given zinc supplementation (Bednorz et al., 2013). The presence of the streptomycin and mercury resistance genes on the Tn21 transposon in avian *E. coli* isolates suggests that both of these genes may co-transfer, which could result in the selection of streptomycin resistance upon exposure to mercuric compounds (Bass et al., 1999).

The bacteria overcome the antibiotic stress using several intrinsic resistant mechanisms such as deactivating enzymes, cell walls, ribosomal modification, and efflux pumps (Sodhi et al., 2021). Latest research has moved to alternative options such as antibacterial based on peptides and nucleic acids, PNAs, Bacteriocins, Bacteriophage therapies (lysins against Gram-positive bacteria), antibodies, anti-virulence compounds such as toxins encoding genes, CRISPR-Cas constructs, and Transition metal complexes are a good candidate for the antimicrobial agents (Aslam et al., 2018).

A wide range of stress, such as the presence of xenobiotics, heavy metals, and antibiotics, are characteristic of marine ecosystems. Microbes can thrive in this environment and get well adapted to environmental stress. The bacterial isolates from this environment are stress-resistant and pollutant-degrading strains. The genus has the capacity for bioremediation and pollutants degradation, such as xenobiotics (azo dye, polyaromatic hydrocarbons, phenols, and phenanthrene), pesticides (Singha et al., 2017; Basharat et al., 2018), and antibiotic degradation (Sodhi et al., 2020c), heavy metal removal (Sodhi et al., 2020a). *A.faecalis* is known to convert the toxic form of arsenic, arsenite, to arsenate (Basharat et al., 2018). Production of nanoparticles has been reported by the genus *Alcaligenes* along with the production of bioplastics and detergents (Basharat et al., 2018).

The previous study by Duran et al., 2019 reported using *Alcaligenes aquatilis* strain QD168 to decontaminate oil-polluted sites (Duran et al., 2019). *Alcaligenes* sp. HPC 1271 showed antimicrobial activity against multi-drug resistant strains *Enterobacter* sp. and *Serratia* sp. GMX1 (Kapley et al., 2016). *Bacilllus subtilis* strain 1556WTNC showed the potential to degrade cephalexin antibiotic and remove 10 mg/L of Cu^2+^, Cd^2+^, Cr^6+^, Ni^2+^, and Zn^2+^ (Al-Gheethi & Ismail, 2014).

A complete genomic sequence of bacteria provides reliable identification and phylogenetic relationship of the bacteria. Whole-genome sequencing (WGS) is a valuable tool for the characterization of the bacterial genome and to study of its biotechnological capabilities. WGS has become a useful tool to monitor the spread of antibiotic resistance, a global threat to healthcare. The WGS aids in producing diagnostics tools and novel antibiotics (Henriksen et al., 2019). The WGS enables us to study bacterial-resistant mechanisms. Ex. *Mycobacterium tuberculosis*, a causative agent of tuberculosis, 454 pyrosequencing helped in the identification of the F0 subunit present in ATP synthase, which acts as a target of bedaquiline, and it became a novel anti-tuberculosis agent (Zhang et al., 2019).

The bacteria in the river Yamuna thrive under the stress of xenobiotics, antibiotics, heavy metals, and an accumulated load of organic matter. In the previous study by the author, the *Alcaligenes* sp. MMA was isolated from the polluted Yamuna (Sodhi et al., 2020b) in M9 minimal media with amoxicillin as a sole carbon source; the bacteria were checked for antibiotic and heavy metal removal efficiency. *Alcaligenes* sp. strain MMA was able to remove 86% of amoxicillin (5 mg/L) in M9 minimal media and showed resistance against multiple heavy metals (Cu^2+^, Cd^2+^, Cr^6+^, Ni^2+^, and Zn^2+^), along with their removal in 72 h. Given this, the current study aims to assess the genomics of the strain MMA and study their structural and functional features to get an insight into their bioremediation potential (Supplementary Figure S1).

## Material and methods

### Isolation of the bacterial strain

Before isolation, bacteria were enriched in sterile M9 minimal media. The media was amended with 5 mg/L of amoxicillin and the concentration was raised subsequently four times (Sodhi et al., 2020c).

### Sequencing of the bacterial genome and assembly

#### DNA extraction and Quality Check

For extracting the genomic DNA from the *Alcaligenes* sp. MMA, DNeasy Blood, and Tissue kit (Qiagen) were used. The extraction was performed according to the kit’s protocol. It was followed by DNA quantification using a nanodrop (ND100- Thermo Fisher) and Qubit 4 Fluorometer (Thermo Fisher). The DNA integrity was checked on 1% agarose gel.

#### Library preparation and sequencing

KAPA HyperPlus kit was used to prepare the sequencing library according to the manufacturer’s protocol. ∼100 ng of DNA input was taken and fragmented enzymatically. The fragments of DNA undergo end repair where the mix converts the overhangs resulting from fragmentation into blunt ends. The 3′ to 5′ exonuclease activity of the end repair mix removes the 3′ overhangs and polymerase activity fills in the 5′ overhangs. To the blunt-ended fragments adenylation is performed by adding a single ‘A’ nucleotide to the 3′ ends. Purification of the samples is done using AMPure beads and further, the DNA is enriched by PCR with 6 cycles using NEBNext Ultra II Q5 master mix, Illumina universal primer, and sample-specific octamer primers. The amplified products are cleaned up by using AMpure beads and the final DNA library was eluted in 15 uLs of 0.1X TE buffer. The fragment analysis was performed on Agilent 2100 Bioanalyzer, by loading 1 uL of the library into Agilent DNA 7500 chip. The sequence used for the whole genome analysis was Illumina Hisq 4000.

#### Bioinformatics analysis

**FIGURE 1:**
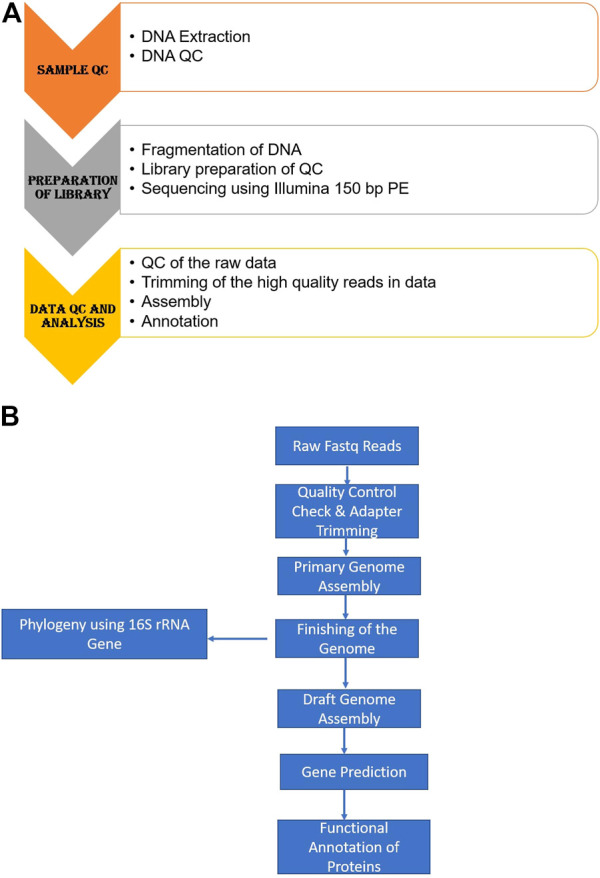
**(A, B)**: Pipeline for the whole genome sequencing of the strain MMA.

The sequenced raw reads are quality-checked using Fastqc (Version 0.11.9) and Multiqc (Version 1.10.1). Once the raw reads pass QC the adapter reads in the samples are trimmed out using Trimgalore (Version 0.6.6). The Trimmed reads are assembled using the *de novo* assembly (Primary assembly) tool called Unicycler. A reference-based assembly (Secondary assembly) was done by downloading the reference genome identified with the help of PubMLST from the NCBI database. The reference-based assembly was done with the CONTIGuator web application (http://contiguator.sourceforge.net/). Figures 1A, B summarize the steps followed in the sequencing of the strain MMA.

### Gene prediction, annotation, and functional characterization of the draft genome

RAST (http:/rast.nmpdr.org/) annotation server with annotation parameters such as “genetic code = 11, E Value cut-off for selection CDSs = 1e-20”. RAST provides high-quality genome annotations for these genomes across the whole phylogenetic tree (Aziz et al., 2008). KEGG was obtained using KEGG automated annotation server (KAAS) and the pie chart was drawn using MS Excel.

### Exploration of ARGs and metal-resistant genes in the draft genome

Antibiotic resistance genes were profiled through the BLAST module of the Antibiotic resistance database (ARDB). ResFinder & comprehensive antibiotic resistance database (CARD) (https://card.mcmaster.ca/analyze/rgi) was used to detect the ARG in the assembled genome (Alcock et al., 2020). ARGs were screened in the Web portal resistant gene identifier (RG1) (v5.1.1), CARD (v3.1.0), and the prediction of the Open Reading Frame is done using Prodigal and homolog detection with the strict, loose, and perfect algorithm. ARGs were studied through the ARDB BLAST module. Metal resistance genes were identified using the BactMet2 database. In the BacMet2 database, blastp was performed to detect the metal resistance genes (Jiang et al., 2021).

### Genome mining to study secondary metabolite in the draft genome

antiSMASH (antibiotics & Secondary Metabolite Analysis Shell) was used to detect the secondary metabolite clusters (Biosynthetic gene clusters) in the draft genome and it was compared with related clusters in other organisms (Weber et al., 2015).

### Data availability

The WGS was submitted to NCBI with BioProject ID-**PRJNA778410** and accession number-**JAJJPP000000000**.

## Results

### Sequencing and functional annotation of the *Alcaligenes* sp. strain MMA

**FIGURE 2:**
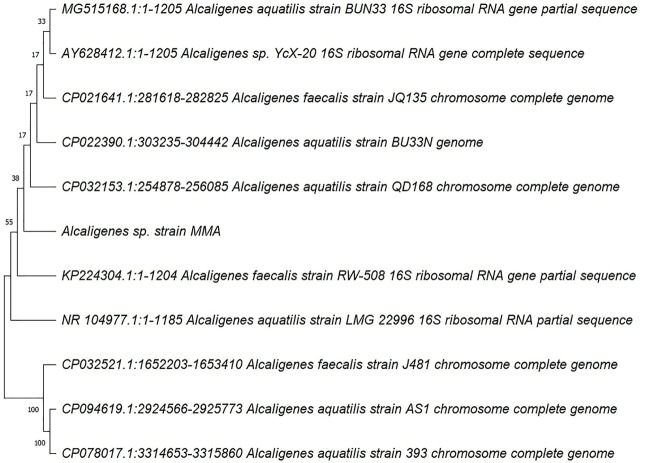
Neighbor-joining tree was constructed using the Maximum Likelihood method with 1000 bootstraps using the software MEGA X.

**FIGURE 3:**
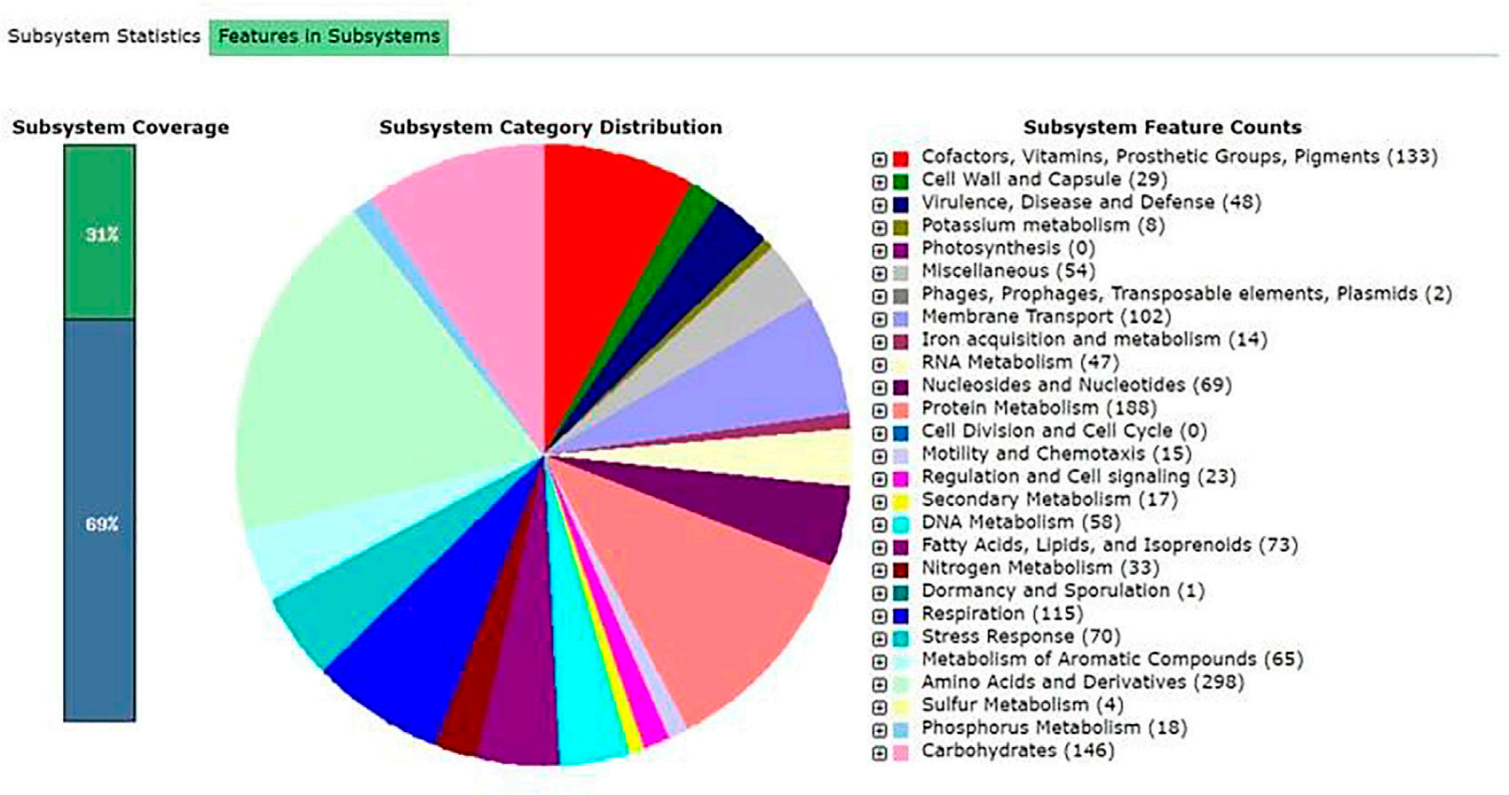
Subsystem distribution in different categories of the bacterium *Alcaligenes* sp. strain MMA. The subsystem coverage shows the total genes present (31% in subsystems and 69% not in subsystems). The *pie chart* indicates the different functions and genes proportions The *numbers shown in parentheses*determines the gene counts with specific functions.

The total length of the genome was 3.9 Mb with a GC content of 55.91%. The assembly resulted in 8 scaffolds and 7 contigs. Supplementary Table S1 shows the assembly statistics of the sequence MMA. Unicycler does the whole genome assembly and the result is a draft assembled fragment of raw data. The multiple alignments were performed and phylogenetic tree was constructed using MEGA X (Figure 1). Figure 2 shows that the MMA is closest to the *Alcaligenes aquatilis* and *Alcaligenes faecalis.* The MMA shows maximum similarity (100% similarity) to the *A. aquatilis* (CP032521.1) *and A. faecalis* (CP032531.1). Genes aiding the bacteria to tolerate stress were present in the *Alcaligenes*. Genes involved in the aromatic compound metabolism were also present in the bacterial strain. Benzoate degradation genes (BenB, BenA, BenC, BenK, BenD, BenF), involved in toluate, catechol, and benzoate degradation are present in the genome (Figure 3). A summary of functional annotation of draft genome MMA using RAST is presented in Supplementary Table S2. Supplementary Table S3 shows the antibiotic and metal-resistant genes in the annotated genome.

COG analysis revealed that the CDS sequences were classified into unknown functions, followed by the genes involved in the transport and metabolism of amino acids and carbohydrates. RAST result shows that the *Alcaligenes* sp. strain MMA genes are mostly involved in the metabolism of organic compounds. The KEGG pathways were obtained from the KAAS server. The KAAS server is involved in the functional annotation of genes by BLAST comparisons against the manually curated KEGG genes databases. The KO (KEGG Orthology) was assigned and the pathways were generated. The result showed that the pathways present in the *Alcaligenes* sp. MMA was majorly the metabolic pathways, i.e., the pathways that are involved in the amino acid synthesis and the biosynthesis of secondary metabolites (Supplementary Figure S2).

### Antibiotic-resistant genes in *Alcaligenes* sp. strain MMA

**FIGURE 4:**
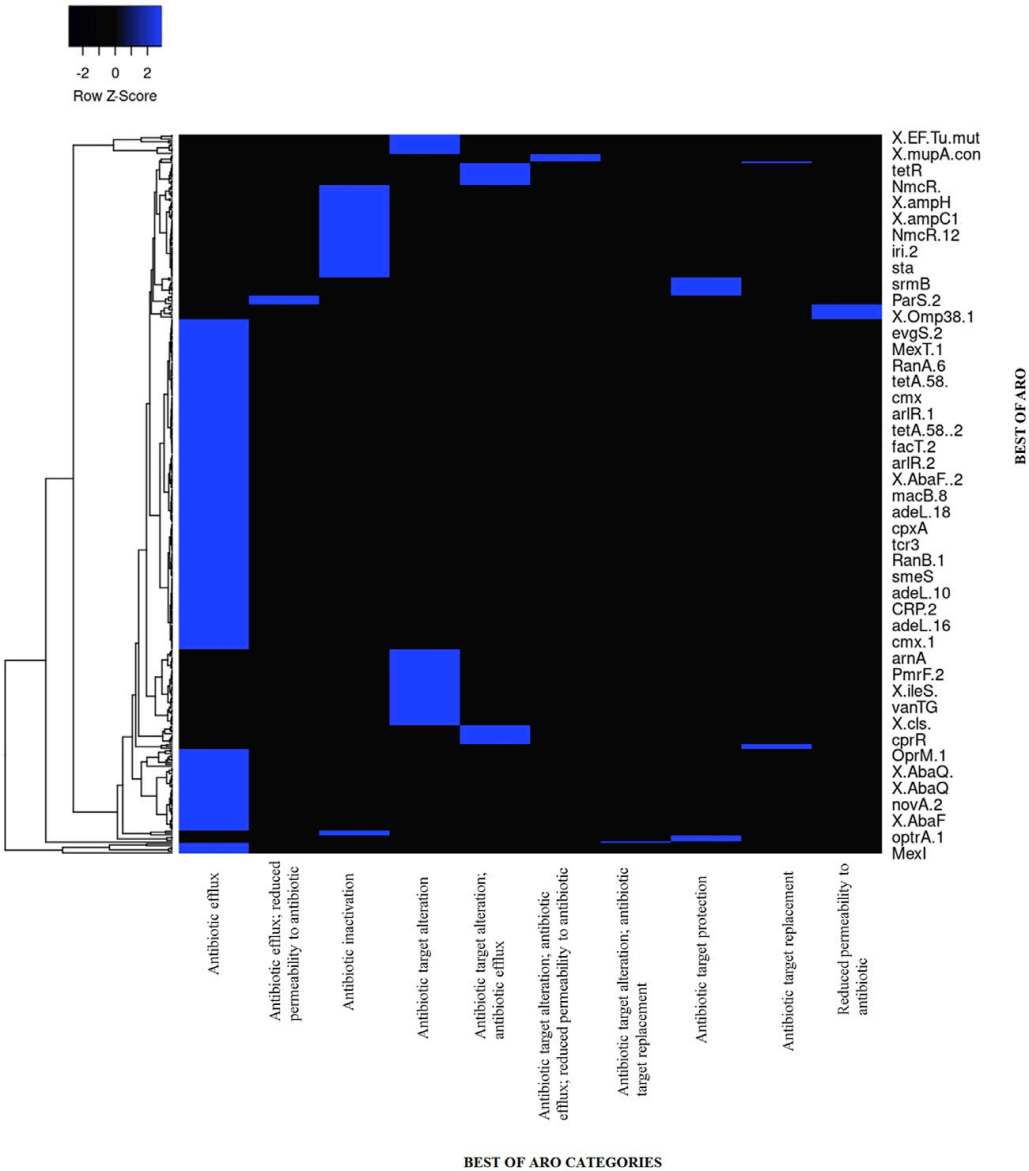
The heat map shows ARO showing similarities of ARGs of our strains with other bacterial species in CARD Similarity of strict matches of ARO are shown in the blue color of other bacterial species and our strain MMA.

**FIGURE 5:**
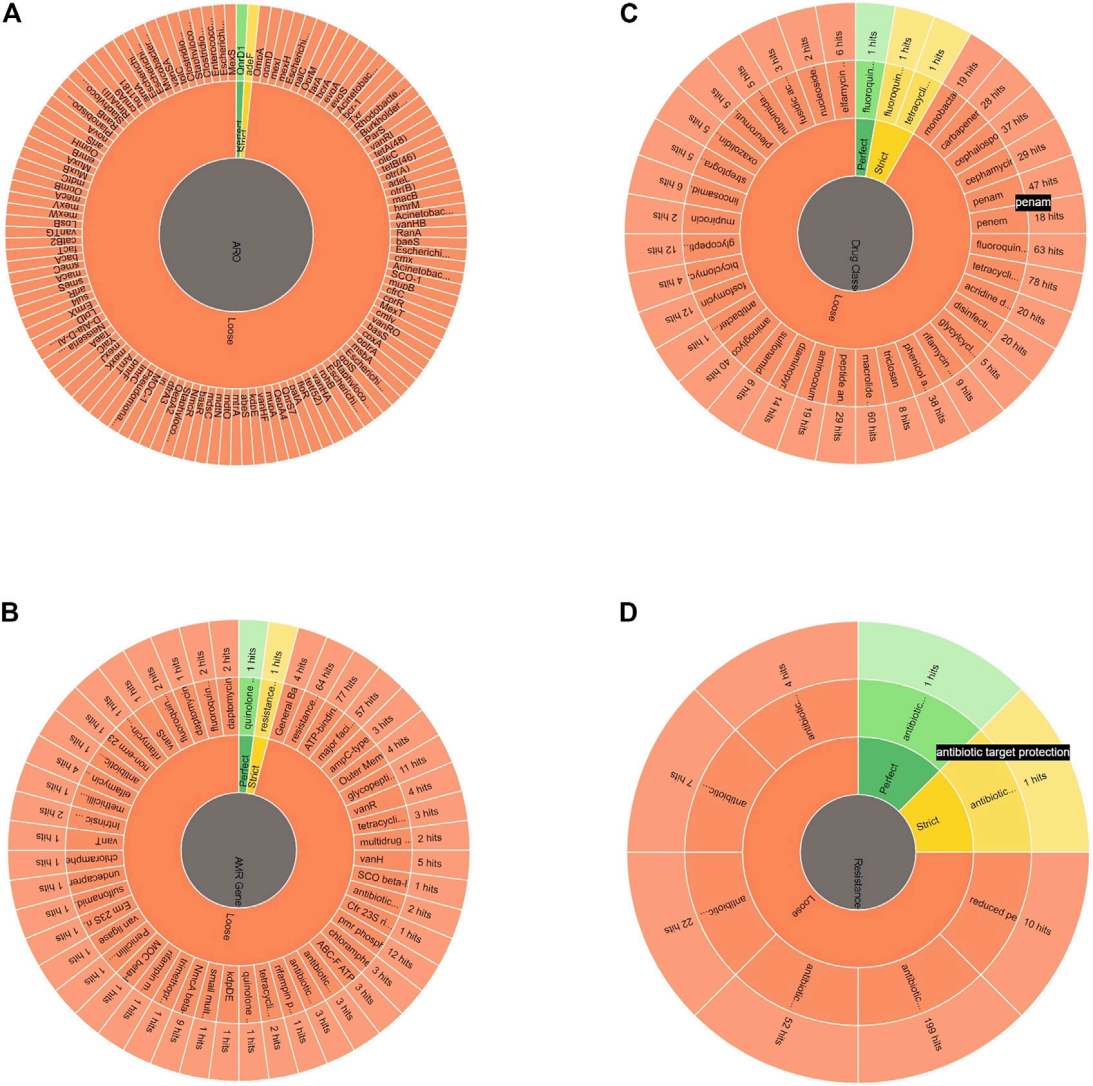
**A)** The depiction of antimicrobial-resistant genes in *Alcaligenes* sp. strain MMA*,* **(B)** The AMR gene family, **(C)** Drug class against which the resistant genes were found and, **(D)** The antibiotic resistance mechanism harbored by the *Alcaligenes* sp. strain MMA.

The *Alcaligenes* sp. MMA showed resistance to tetracycline, fluoroquinolones, beta-lactams, penams, and macrolides. A heat map showing the AROs was drawn. A total of 279 loose hits along with 1 perfect and strict hit were found. Sequences with lower cut-off values but high blast similarity depicted resistance to tetracycline, fluoroquinolones, beta-lactams, penams, and macrolides. The heat map shows ARO showing similarities of ARGs of our strains with other bacterial species in CARD (Figure 4; Table 1). The assembled MMA genome was used as an input in RGI, and a wheel chart depicting the AMR genes, drug, and class was obtained (Figure 5). Figure 5, shows the distribution of antimicrobial resistance genes in the *Alcaligenes* sp. MMA genome. The *Alcaligenes* sp. strain MMA harbor genes providing resistance to fluoroquinolones, tetracyclines, penams, rifamycin, and sulfonamides along with that the resistance mechanisms mainly antibiotic efflux, reduced permeability, and antibiotic target alteration.

**TABLE 1:** Antibiotic-resistant genes present in *Alcaligenes* sp. strain MMA.

S.No.	Criteria used by RG1	ARO term	Family of AMR genes	Drug class	Mechanism of resistance	% Identity with the matching region
1	Perfect	QnrD1	Quinolone resistance protein	Fluoroquinolones	Antibiotic target protection	100
2	Strict	AdeF	Efflux pump and Resistance-nodulation-cell division (RND)	Fluoroquinolones and tetracyclines	Efflux of antibiotics	42.6
.3	Loose	OmpA	Porins with reduced permeability to beta-lactams	Monobactam, cephalosporin, cephamycin, penam, penem carbapenem	Reduced permeability to antibiotics	40.26
4	Loose	OpmD	RND and efflux pump	Fluoroquinolone and tetracyclines	Efflux of antibiotics	48.12
5	Loose	MexL	RND and efflux pump	Fluoroquinolones and tetracyclines	Efflux of antibiotics	76.18
6	Loose	*Escherichia coli* soxR mutation conferring antibiotic resistance	RND, efflux pump	Penam, rifamycin, fluoroquinolones, and tetracyclines	Alteration of target of antibiotics and efflux of antibiotics	62.59
7	Loose	OprM	RND and efflux pump	Penam, phenol, sulfonamide, and tetracyclines	Efflux of antibiotics	43.56

#### Heavy metal resistance gene finder

Bactmet2 was used to check for the heavy metal resistance genes in our genome and various genes which are responsible for providing resistance against toxic metals were found. Cadmium and lead transcription regulator genes are present along with the multidrug efflux transporter genes are present further suggesting that the bacterial strain is responsible for metal resistance (Rutledge and Challis, 2015). Genes encoding resistance to multiple heavy metals such as Cd, Ni, Cu, and Zn are present in the genome of the strain MMA. The results are following our previous study in which the strain MMA was able to remove the heavy metals Cu, Cr, Cd, Ni, and Zn. Table 2 and Supplementary Figure S3, show the presence of heavy metal resistance genes in *Alcaligenes* sp. strain MMA. The blastp results of the heavy metal-resistant genes found in the draft genome are shown in Supplementary Table S4.

**TABLE 2:** Heavy metal resistance genes in *Alcaligenes* sp. strain MMA.

S.No.	Genes	Count of genes
1	molybdate ABC transporter permease subunit [*Alcaligenes faecalis*] [*modB*]	4
2	Cd(II)/Pb(II) responsive transcriptional regulator [*Pseudomonas putida*] [*cadR*]	3
3	molybdate ABC transporter protein [*Alcaligenes faecalis*] [*modA*]	3
4	ABC transporter [*Vibrio shilonii*] [*vcaM*]	2
5	aconitate hydratase [*Mycobacterium gordonae*] [*acn*]	2
6	aconitate hydratase [*Mycobacterium kumamotonense*] [*acn*]	2
7	arsenical resistance protein ArsH [*Variovorax paradoxus*] [*arsH*]	2
8	Bcr/CflA family drug resistance efflux transporter [*Escherichia coli*] [*bcr*]	2
9	Bcr/CflA family multidrug efflux MFS transporter [*Escherichia coli*] [*bcr*]	2
10	MULTISPECIES: superoxide dismutase [Fe] [*Ralstonia s*p.] [*sodB*]	2
11	nickel ABC transporter permease subunit NikB [*Stappia indica*] [*nikB*]	2
12	aconitate hydratase AcnA [*Mycobacterium* sp. ACS4331] [*acn*]	1
13	arsenical resistance protein ArsH [*Bordetella* sp. SCN 67-23] [*arsH*]	1
14	bacterioferritin [*Actinomadura madurae*] [*bfrA*]	1
15	cation acetate symporter [*Pseudomonas massiliensis*] [*actP*]	1
16	cation acetate symporter [*Wolinella succinogenes*] [*actP*]	1
18	Co2+/Mg2+ efflux protein ApaG [*Escherichia coli*] [*corD*]	1
19	DNA-binding response regulator [*Burkholderia thailandensis*] [*irlR*]	1
20	enoyl-ACP reductase FabI [*Budvicia aquatica*] [*fabI*]	1
21	enoyl-ACP reductase FabI [*Pragia fontium*] [*fabI*]	1
22	LysR family transcriptional regualator [*Herbaspirillum rhizosphaerae*] [*adeL*]	1
23	LysR family transcriptional regulator [*Paraburkholderia diazotrophica*] [*adeL*]	1
24	LysR family transcriptional regulator [*Paraburkholderia sartisoli*] [*adeL*]	1
25	LysR family transcriptional regulator [*Rhodocyclaceae* bacterium Paddy-1] [*adeL*]	1
26	MULTISPECIES: molybdate ABC transporter permease subunit [*Alcaligenes* sp.] [*modB*]	1
27	MULTISPECIES: superoxide dismutase [*Bordetella* sp.] [*sodA*]	1
28	nickel ABC transporter permease subunit NikB [*Afifella marina*] [*nikB*]	1
29	phosphate transporter permease subunit PtsA [*Escherichia coli*] [*pstA*]	1
30	PREDICTED: uncharacterized protein LOC102318278 [*Pantholops hodgsonii*] [*arsH*]	1
31	superoxide dismutase [*Cupriavidus pauculus*] [*sodB*]	1
32	superoxide dismutase [Fe] [*Beggiatoa* sp. 4572_84] [*sodB*]	1
33	transcriptional regulator, LysR family [*Methylobacillus flagellatus* KT] [*adeL*]	1

### Secondary metabolite analysis

Secondary metabolite clusters are present in the draft genome, namely, T1PKS, Non-ribosomal peptides (NRPS), resorcinol, terpene, ectoine, beta lactone, and phosphonate. Among these, two clusters have high homology to biosynthetic gene clusters (BGCs) encoding ectoine (75% similarity) and NRPS (60% similarity to Bacillibactin) Table 3.

**TABLE 3:** BGCs in the *Alcaligenes* sp. strain MMA.

S.No.	Region	Type	From	To	Most similar BGC	Identity (%)
1	*Region 1*	T1PKS	553,828	601,402	Emulsan	9
2	*Region 2*	NRPS, resorcinol	864,811	940,561	Bacillibactin	60
3	*Region 3*	Terpene	1,710,809	1,732,539	Burkholderic acid	13
4	*Region 4*	Ectoine	2,180,435	2,190,827	Ectoine	75
5	*Region 5*	Betalactone	3,113,637	3,139,925	Quinolobactin	20
6	*Region 6*	Betalactone	3,629,780	3,657,913		
7	*Region 7*	Phosphonate	3,821,363	3,862,241		

## Discussion

In the previous studies by the author Sodhi et al., 2020a, and Sodhi et al., 2020b, a potent amoxicillin and multiple heavy metal-resistant strain MMA was isolated from the contaminated river Yamuna. The ability of the organism to tolerate micropollutants was revealed through sequencing, and the gene machinery responsible for these characteristics were identified and is currently the subject of more *in vitro* and silico research. Our isolate possessed amoxicillin degradation and multiple heavy metals removal abilities as well as micropollutant resistance. It has many genes for metal transport and sensing, allowing it to develop a metal homeostasis system that aids in survival and growth in contaminated environments. A deeper understanding of the bacterium’s metal homeostasis could be obtained through more research such as differentiating expression patterns and examining how the proteome changes in response to metal stress. Understanding the remediation and eco-friendly qualities of *Alcaligenes* sp. MMA requires the completion of its genome. Since the antibiotic resistance mechanism affects metal homeostasis and *vice versa*, a large number of antibiotic resistance genes have been mined.

Initially, the 16SrRNA-based phylogenetics identified the strain as *Alcaligenes* sp. MMA was able to degrade amoxicillin and remove multiple heavy metals. Whole genome sequencing was carried out which showed the genomic properties of the bacteria regarding the presence of antibiotic and heavy metal resistance genes. The genus *Alcaligenes* is known to have biotechnological applications and degradation capabilities (Chain et al., 2009). Antibiotic and heavy metals contamination is increasing worldwide. Antibiotic resistance is a serious threat to the entire nation leading to the generation of superbugs. The *Alcaligenes* sp. MMA is resistant to amoxicillin which makes the bacteria an opportunistic pathogen. The major concerns regarding this opportunistic pathogen are that it can spread in the hospital environment, causing nosocomial infections, and can disseminate antibiotic resistance when combined with acquired antibiotic-resistant genes and their intrinsic mechanisms (Kumar et al., 2022). Moreover, the strain was isolated from the polluted Yamuna, which has a prevalence of fluoroquinolones and beta-lactams (Velpandian et al., 2018; Sodhi et al., 2020a). *Alcaligenes* sp. strain MMA was able to remove 86% of amoxicillin (5 mg/L) in M9 minimal media and showed resistance against multiple heavy metals (Copper Cu^2+^, Cadmium Cd^2+^, Chromium Cr^6+^, Nickel Ni^2+,^ and Zinc Zn^2+^), along with their removal in 72 h. The genome of strain MMA was sequenced which resulted in a draft genome of 3.9 Mb. This bacterium has a major role in the bioremediation of contaminants. The RAST analysis showed the genes involved in the biosynthesis of co-factors and involved in protein metabolism. RAST is an automated server that computes the similarities of the uploaded genome to the genomes already present in the SEED database. Nineteen genes are involved in denitrification, and 14 are involved in the ammonia assimilation pathway and nitrosative stress. They possess the ability to act as a denitrifying organism. Glutamine synthetase- I (GS-I), GS-III along with transcription regulatory genes (NarR), and nitrate/nitrite transporter (NarK) are present. NirV and Nir K (nitrite reduction accessory genes) are also present. Benzoate degradation genes (BenB, BenA, BenC, BenK, BenD, BenF) involved in the degradation of toluate, catechol, and benzoate are present in the genome. The results are in accordance with the previous studies of Basharat et al., 2018, in which they identified hydrocarbon-degrading gene clusters. The bacterial strain *Alcaligenes* sp. strain MMA has genes and pathways involved in the metabolism of aromatic compounds and hydrocarbons which means that the bacteria and their enzymes might be used for the degradation of xenobiotics and other aromatic compounds. The bacteria can adapt to harsh environmental conditions and be isolated from a polluted river Yamuna where there are known concentrations of xenobiotics such as pesticides (Kaushik et al., 2008). COG analysis revealed that the CDS sequences were classified into unknown functions, followed by the genes involved in the transport and metabolism of amino acids and carbohydrates. RAST result shows that the *Alcaligenes* sp. strain MMA genes are mostly involved in the metabolism of organic compounds. Bacterial growth is dependent mostly on carbon and nitrogen sources so most of the functional genes in the bacteria are devoted to organic acid biosynthesis and metabolism.

Increasing antibiotic consumption has become a cause of grave concern. Bacteria are avid producers of antibiotics and can resist them for their survival. Horizontal gene transfer (HGT) is one way of the dissemination of ARGs in antibiotic susceptible strains. The bacteria were isolated from the contaminated river Yamuna. Many studies including Velpandian et al., 2018; Sodhi et al., 2020b showed the occurrence of antibiotics in the Yamuna. Therefore, the ARGs of *Alcaligenes* sp. strain MMA were cataloged to understand the environmental reservoir of such genes. CARD consists of antibiotic-resistant ontology (ARO) for the classification of ARG data (Jia et al., 2016). Ontologies are controlled vocabularies and an integral part of genomics and bioinformatics, important as they aid in the robust investigation of data. For this reason, the antibiotic-resistant profiling of our strain was done using RGI in CARD. The *Alcaligenes* sp. MMA showed resistance to tetracycline, fluoroquinolones, beta-lactams, penams, and macrolides. Genes encoding resistance to multiple heavy metals such as Cd, Ni, Cu, and Zn are present in the genome of the strain MMA. The results are following our previous study in which the strain MMA was able to remove the heavy metals Cu, Cr, Cd, Ni, and Zn.

The secondary metabolites of fungi and bacteria are a rich source of novel bioactive compounds which have a potential application in the pharmaceutical industry such as in new drug candidates such as antibiotics, cholesterol-lowering drugs as well as anti-tumor drugs (Nikaido, 2009). antiSMASH is a comprehensive pipeline that aids in the identification of biosynthetic loci (comprising of terpins, beta-lactams, polyketides, aminoglycosides, beta-lactams, lantibiotics, aminocoumarins, bacteriocins, siderophores, melanin among others). Since the use of antibiotics has resulted in the development of antimicrobial resistance in pathogens, there is always a need for new drug candidates for addressing the problem of antibiotic resistance (Abdel-Razek et al., 2020; Quinn et al., 2020). *Alcaligenes* sp. strain MMA genome revealed its potential for the production of bioactive compounds such as secondary metabolites. The NRPs are synthesized naturally by microbes such as fungi, bacteria, and eukaryotic symbionts. The natural products of NRPs include antibiotics (such as vancomycin, penicillin, actinomycin, and cephalosporin), also bleomycin which is cytotoxic. Bacillibactin is a siderophore, bacteria synthesize siderophores to enhance the bioremediation of heavy metals and recently the cefiderocol (Fetroja) was approved by US FDA as a siderophore cephalosporin which binds to the penicillin-binding protein 3 thereby inhibiting the cell wall of the bacteria and the siderophore binds to free iron and gain additional cell entry**.**

## Conclusion

**FIGURE 6:**
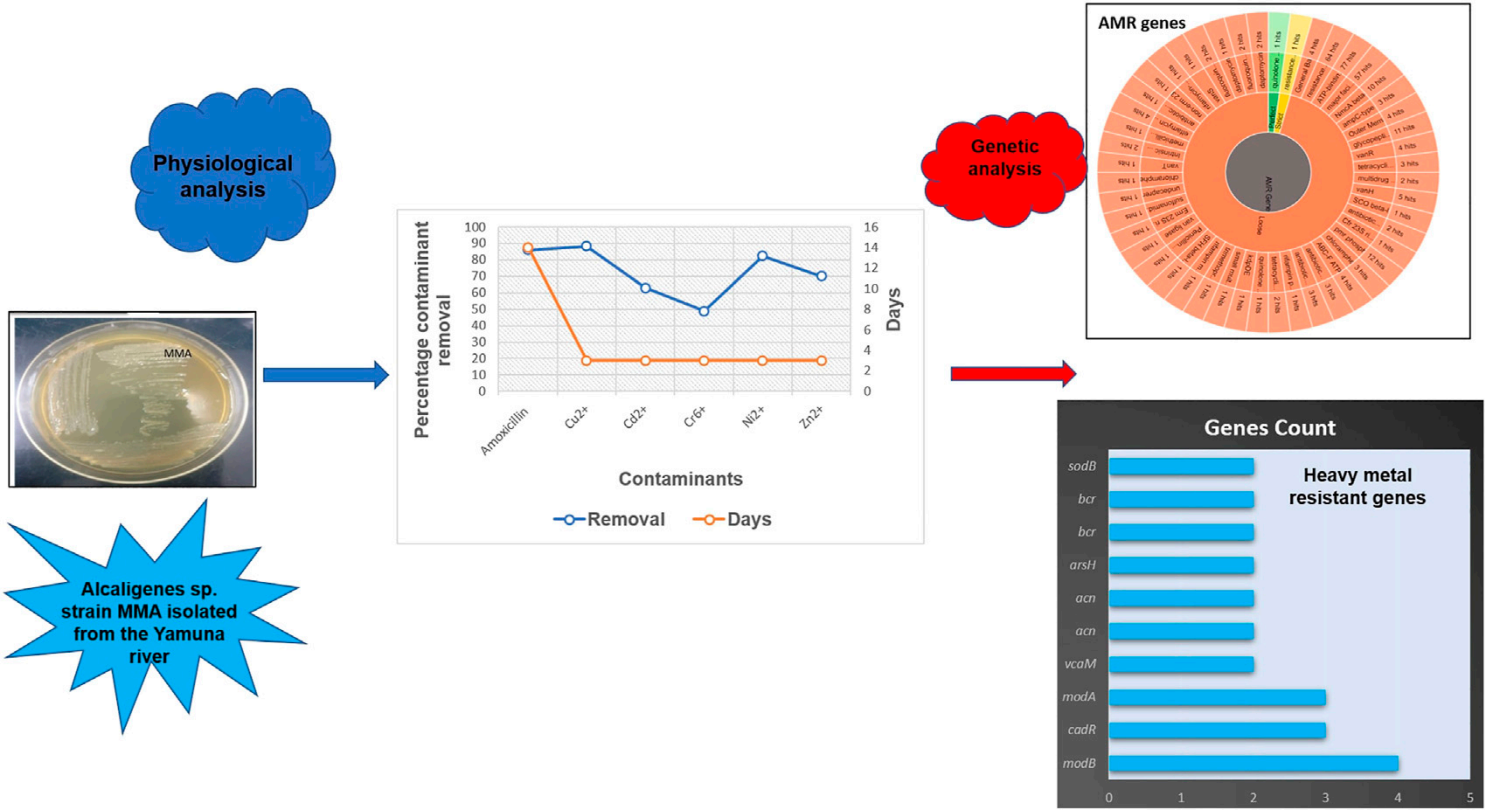
*Alcaligenes* sp. strain MMA physiological analysis linked with the genetic analysis.

*Alcaligenes* sp. strain MMA was sequenced and assembled to study its structural and functional traits. The strain was isolated and showed the potential to remove the amoxicillin and multiple heavy metals in author’s previous studies (Supplementary Figure S1). So, to shed the light on the genome, the strain MMA was sequenced and resulted in a draft genome of 3.9 Mb. The genome annotation was done using RAST and revealed the presence of 3,685 protein-coding genes. Because of the spread of antimicrobial resistance, and generation of multi-drug resistant pathogens (MDR), the strain MMA was checked for the potential ARGs and, also for the heavy metal resistance genes which sheds the light on the bioremediation capabilities of the genome. Further, we checked for the presence of BGCs in the draft genome and many types of BGCs such as siderophore and ectoine were predicted. The results of this study provide information on the strain MMA genome and are valuable for the researcher in further exploitation of the strain MMA for bioremediation. This study marks the potential of the isolated bacterial strain in the removal of heavy metals and antibiotics and an eco-friendly bacterium that can aid in research related to the environment. Research on the xenobiotic-metabolizing bacterial isolates aids in exploiting their biodegradative potential and helps to redeem sites that would remain contaminated otherwise and are expensive to be reclaimed by other technology. The WGS enables the researchers to explore various enzymes which can be used sustainably for environmental clean-up and also mark the production of novel antimicrobial compounds for the surveillance of antimicrobial resistance (Figure 6).

## Data availability statement

The datasets presented in this study can be found in online repositories. The names of the repository/repositories and accession number(s) can be found below: NCBI with BioProject ID-PRJNA778410 and accession number JAJJPP000000000.

## Supplementary material

SUPPLEMENTARY FIGURE S1 Amoxicillin and heavy metal removal by Alcaligenes sp. MMA (Sodhi et al., 2020a & Sodhi et al., 2020b).

SUPPLEMENTARY FIGURE S2 KEGG pathways obtained from KAAS server.

SUPPLEMENTARY FIGURE S3 Metal resistance genes in Alcaligenes sp. strain MMA.
