# Supplementary material for: Whole-genome sequencing of Alcaligenes sp. strain MMA: insight into the antibiotic and heavy metal resistant genes
Source: Front Pharmacol. 2023 May 11;14:1144561. doi: 10.3389/fphar.2023.1144561 (PMC10213877; doi:10.3389/fphar.2023.1144561)
Supplement: Supplementary file 1 [file Table1.DOCX]

**Supplementary Table 1:** Assembly statistics of the sequence MMA

| **Sample** | **Total bases** | **Average** | **N50 (bp)** | **GC content (%)** |
| --- | --- | --- | --- | --- |
| MMA | 3944519 | 493064.88 | 906161 | 55.91 |
