# Supplementary figures and images for: Whole-genome sequencing of Alcaligenes sp. strain MMA: insight into the antibiotic and heavy metal resistant genes
Source: Front Pharmacol. 2023 May 11;14:1144561. doi: 10.3389/fphar.2023.1144561 (PMC10213877; doi:10.3389/fphar.2023.1144561)

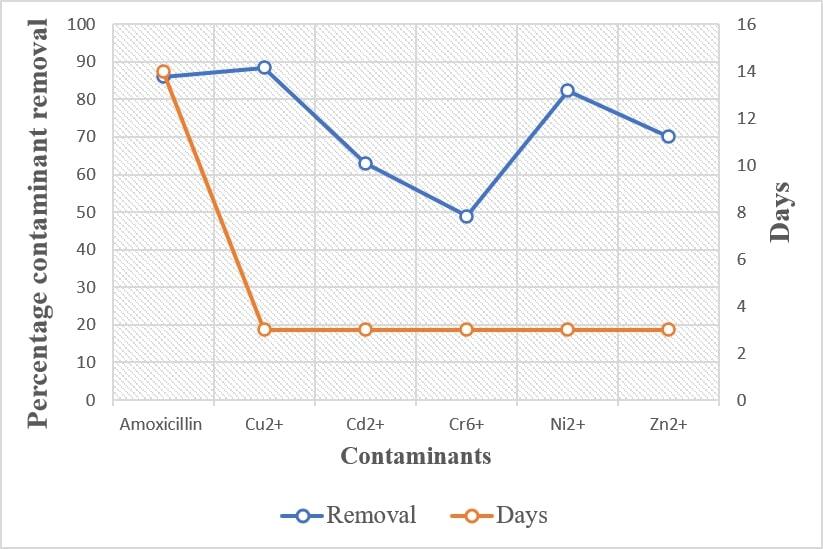

Supplement: Supplementary file 2 [file Image1.JPEG]

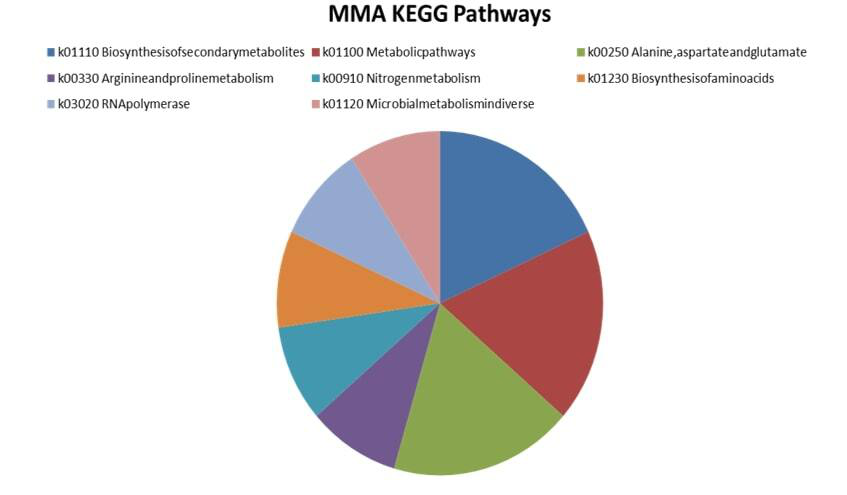

Supplement: Supplementary file 5 [file Image2.PNG]

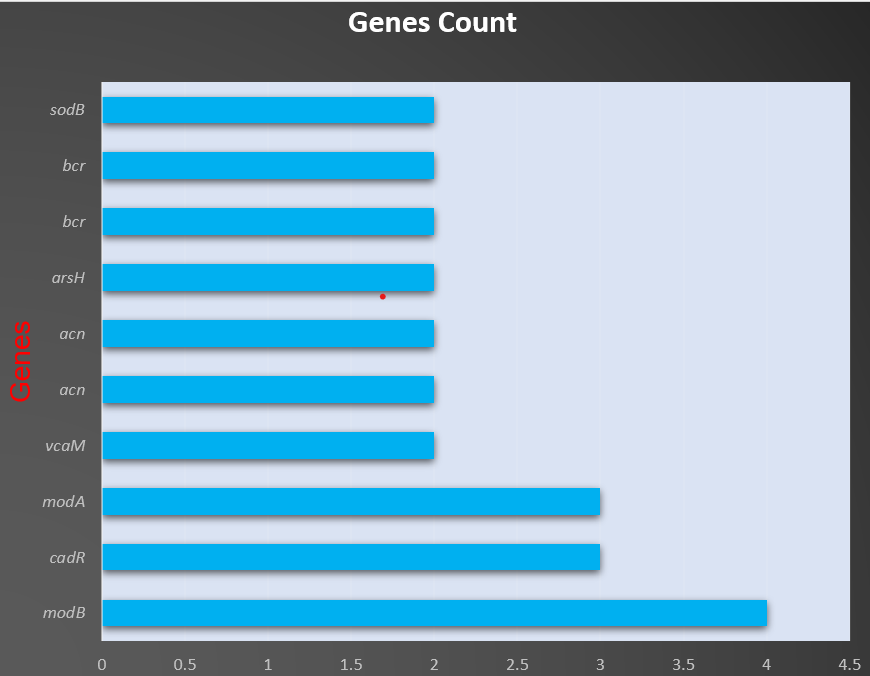

Supplement: Supplementary file 7 [file Image3.PNG]
