# Supplementary material for: Whole-genome sequencing of Alcaligenes sp. strain MMA: insight into the antibiotic and heavy metal resistant genes
Source: Front Pharmacol. 2023 May 11;14:1144561. doi: 10.3389/fphar.2023.1144561 (PMC10213877; doi:10.3389/fphar.2023.1144561)
Supplement: Supplementary file 3 [file Table2.docx]

**Supplementary Table 2:** Summary of functional annotation of draft genome MMA using RAST

| **Metric** | **MMA draft genome** |
| --- | --- |
| Number of predicted genes | 3740 |
| Number of genes coding for protein | 3685 |
| Number of characterized protein | 2837 |
| Number of hypothetical/putative protein | 848 |
