# Supplementary material for: Whole-genome sequencing of Alcaligenes sp. strain MMA: insight into the antibiotic and heavy metal resistant genes
Source: Front Pharmacol. 2023 May 11;14:1144561. doi: 10.3389/fphar.2023.1144561 (PMC10213877; doi:10.3389/fphar.2023.1144561)
Supplement: Supplementary file 4 [file Table3.docx]

**Supplementary table 3**: The antibiotic and metal resistant genes in the annotated genome

| **contig_id** | **feature_id** | **type** | **Location** | **start** | **stop** | **strand** | **Function** |
| --- | --- | --- | --- | --- | --- | --- | --- |
| Scaffold_1 | [fig\|323284.9.peg.50](http://rast.nmpdr.org/seedviewer.cgi?page=Annotation&feature=fig\|323284.9.peg.50) | Protein Encoding Gene | Scaffold_1_46574_47464 | 46574 | 47464 | + | Permease of the drug/metabolite transporter (DMT) superfamily |
| Scaffold_1 | [fig\|323284.9.peg.107](http://rast.nmpdr.org/seedviewer.cgi?page=Annotation&feature=fig\|323284.9.peg.107) | Protein Encoding Gene | Scaffold_1_112771_110876 | 112771 | 110876 | - | ABC transporter, permease protein 1 (cluster 4, leucine/isoleucine/valine/benzoate) / ABC transporter, permease protein 2 (cluster 4, leucine/isoleucine/valine/benzoate) |
| Scaffold_1 | [fig\|323284.9.peg.110](http://rast.nmpdr.org/seedviewer.cgi?page=Annotation&feature=fig\|323284.9.peg.110) | Protein Encoding Gene | Scaffold_1_114528_116963 | 114528 | 116963 | + | Penicillin G acylase precursor (EC 3.5.1.11) |
| Scaffold_1 | [fig\|323284.9.peg.293](http://rast.nmpdr.org/seedviewer.cgi?page=Annotation&feature=fig\|323284.9.peg.293) | Protein Encoding Gene | Scaffold_1_316822_315677 | 316822 | 315677 | - | Copper-containing nitrite reductase (EC 1.7.2.1) |
| Scaffold_1 | [fig\|323284.9.peg.319](http://rast.nmpdr.org/seedviewer.cgi?page=Annotation&feature=fig\|323284.9.peg.319) | Protein Encoding Gene | Scaffold_1_346005_344530 | 346005 | 344530 | - | Efflux transport system, outer membrane factor (OMF) lipoprotein |
| Scaffold_1 | [fig\|323284.9.peg.320](http://rast.nmpdr.org/seedviewer.cgi?page=Annotation&feature=fig\|323284.9.peg.320) | Protein Encoding Gene | Scaffold_1_349116_346012 | 349116 | 346012 | - | Multidrug efflux system MdtABC-TolC, inner-membrane proton/drug antiporter MdtC (RND type) |
| Scaffold_1 | fig\|323284.9.peg.3175 | Protein Encoding Gene | Scaffold_1_338069_3382009 | 338069 | 3382009 | + | Mg/Co/Ni transporter MgtE, CBS domain-containing |
| Scaffold_1 | [fig\|323284.9.peg.867](http://rast.nmpdr.org/seedviewer.cgi?page=Annotation&feature=fig\|323284.9.peg.867) | Protein Encoding Gene | Scaffold_1_937082_937753 | 937082 | 937753 | + | ABC polar amino acid transporter, inner membrane subunit |
| Scaffold_1 | [fig\|323284.9.peg.1417](http://rast.nmpdr.org/seedviewer.cgi?page=Annotation&feature=fig\|323284.9.peg.1417) | Protein Encoding Gene | Scaffold_1_1534493_1535671 | 1534493 | 1535671 | + | Macrolide-specific efflux protein MacA |
| Scaffold_1 | [fig\|323284.9.peg.1418](http://rast.nmpdr.org/seedviewer.cgi?page=Annotation&feature=fig\|323284.9.peg.1418) | Protein Encoding Gene | Scaffold_1_1535665_1537623 | 1535665 | 1537623 | + | Macrolide export ATP-binding/permease protein MacB |
| Scaffold_1 | [fig\|323284.9.peg.1421](http://rast.nmpdr.org/seedviewer.cgi?page=Annotation&feature=fig\|323284.9.peg.1421) | Protein Encoding Gene | Scaffold_1_1541749_1539905 | 1541749 | 1539905 | - | Efflux ABC transporter, permease/ATP-binding protein Atu2242 |
| Scaffold_1 | [fig\|323284.9.peg.1762](http://rast.nmpdr.org/seedviewer.cgi?page=Annotation&feature=fig\|323284.9.peg.1762) | Protein Encoding Gene | Scaffold_1_1923552_1924757 | 1923552 | 1924757 | + | Multidrug resistance transporter, Bcr/CflA family |
| Scaffold_1 | [fig\|323284.9.peg.1763](http://rast.nmpdr.org/seedviewer.cgi?page=Annotation&feature=fig\|323284.9.peg.1763) | Protein Encoding Gene | Scaffold_1_1924874_1927069 | 1924874 | 1927069 | + | Putative OMR family iron-siderophore receptor precursor |
| Scaffold_1 | [fig\|323284.9.peg.2183](http://rast.nmpdr.org/seedviewer.cgi?page=Annotation&feature=fig\|323284.9.peg.2183) | Protein Encoding Gene | Scaffold_1_2387044_2385743 | 2387044 | 2385743 | - | NICKEL-COBALT-CADMIUM RESISTANCE PROTEIN NCCN |
| Scaffold_1 | [fig\|323284.9.peg.3542](http://rast.nmpdr.org/seedviewer.cgi?page=Annotation&feature=fig\|323284.9.peg.3542) | Protein Encoding Gene | Scaffold_1_3782103_3781042 | 3782103 | 3781042 | - | Beta-lactamase class C-like and penicillin binding proteins (PBPs) superfamily |
| Scaffold_1 | [fig\|323284.9.peg.3543](http://rast.nmpdr.org/seedviewer.cgi?page=Annotation&feature=fig\|323284.9.peg.3543) | Protein Encoding Gene | Scaffold_1_3784637_3782103 | 3784637 | 3782103 | - | Ferrichrome-iron receptor |
