# Supplementary material for: Whole-genome sequencing of Alcaligenes sp. strain MMA: insight into the antibiotic and heavy metal resistant genes
Source: Front Pharmacol. 2023 May 11;14:1144561. doi: 10.3389/fphar.2023.1144561 (PMC10213877; doi:10.3389/fphar.2023.1144561)
Supplement: Supplementary file 6 [file Table4.docx]

**Supplementary Table 4**: Heavy metal resistant genes identified using blastp of the Bactmet2

| **S. No.** | **Genes** | **% Identity** |
| --- | --- | --- |
| 1. 1 | enoyl-ACP reductase FabI [Budvicia aquatica] [fabI] | 60.536 |
|  | enoyl-ACP reductase FabI [Pragia fontium] [fabI] | 59.77 |
|  | PREDICTED: uncharacterized protein LOC102318278 [Pantholops hodgsonii] [arsH] | 40.741 |
|  | arsenical resistance protein ArsH [Bordetella sp. SCN 67-23] [arsH] | 76.639 |
|  | arsenical resistance protein ArsH [Variovorax paradoxus] [arsH] | 77.049 |
|  | arsenical resistance protein ArsH [Variovorax paradoxus] [arsH] | 76.23 |
|  | two-component system response regulator BasR [Serratia fonticola] [pmrA] | 42.342 |
|  | DNA-binding response regulator [Burkholderia thailandensis] [irlR] | 38.428 |
|  | aconitate hydratase AcnA [Mycobacterium sp. ACS4331] [acn] | 68.471 |
|  | aconitate hydratase [Mycobacterium gordonae] [acn] | 67.55 |
|  | aconitate hydratase [Mycobacterium gordonae] [acn] | 67.371 |
|  | Co2+/Mg2+ efflux protein ApaG [Escherichia coli] [corD] | 45.968 |
|  | LysR family transcriptional regulator [Rhodocyclaceae bacterium Paddy-1] [adeL] | 49.164 |
|  | transcriptional regulator, LysR family [Methylobacillus flagellatus KT] [adeL] | 48.495 |
|  | LysR family transcriptional regulator [Paraburkholderia sartisoli] [adeL] | 49.164 |
|  | LysR family transcriptional regulator [Paraburkholderia diazotrophica] [adeL] | 47.826 |
|  | LysR family transcriptional regulator [Herbaspirillum rhizosphaerae] [adeL] | 45.485 |
|  | molybdate ABC transporter substrate-binding protein [Alcaligenes faecalis] [modA] | 95.582 |
|  | molybdate ABC transporter substrate-binding protein [Alcaligenes faecalis] [modA] | 95.582 |
|  | molybdate ABC transporter substrate-binding protein [Alcaligenes sp. EGD-AK7] [modA] | 95.582 |
|  | molybdate ABC transporter substrate-binding protein [Alcaligenes faecalis] [modA] | 95.181 |
|  | MULTISPECIES: molybdate ABC transporter substrate-binding protein [Alcaligenaceae] [modA] | 95.181 |
|  | molybdate ABC transporter permease subunit [Alcaligenes faecalis] [modB] | 100 |
|  | molybdate ABC transporter permease subunit [Alcaligenes faecalis] [modB] | 96.46 |
|  | MULTISPECIES: molybdate ABC transporter permease subunit [Alcaligenes] [modB] | 96.46 |
|  | molybdate ABC transporter permease subunit [Alcaligenes faecalis] [modB] | 95.133 |
|  | molybdate ABC transporter permease subunit [Alcaligenes faecalis] [modB] | 96.018 |
|  | phosphate transporter permease subunit PtsA [Escherichia coli] [pstA] | 55.326 |
|  | phosphate ABC transporter ATP-binding protein PstB [Bordetella ansorpii] [pstB] | 91.892 |
|  | phosphate ABC transporter ATP-binding protein PstB [Bordetella genomosp. 1] [pstB] | 90.347 |
|  | MULTISPECIES: ABC transporter ATP-binding protein [Vibrio] [vcaM] | 56.29 |
|  | ABC transporter ATP-binding protein [Vibrio shilonii] [vcaM] | 56.29 |
|  | ABC transporter ATP-binding protein [Vibrio shilonii] [vcaM] | 56.29 |
|  | nickel ABC transporter permease subunit NikB [Afifella marina] [nikB] | 26.074 |
|  | MULTISPECIES: nickel ABC transporter permease subunit NikB [Citreicella] [nikB] | 26.687 |
|  | nickel ABC transporter permease subunit NikB [Stappia indica] [nikB] | 26.38 |
|  | nickel ABC transporter permease subunit NikB [Stappia indica] [nikB] | 26.074 |
|  | Bcr/CflA family multidrug efflux MFS transporter [Escherichia coli] [bcr] | 36.649 |
|  | MULTISPECIES: Bcr/CflA family multidrug efflux MFS transporter [Escherichia] [bcr] | 36.649 |
|  | Bcr/CflA family drug resistance efflux transporter [Escherichia coli] [bcr] | 36.387 |
|  | Bcr/CflA family multidrug efflux MFS transporter [Escherichia coli] [bcr] | 36.649 |
|  | Bcr/CflA family drug resistance efflux transporter [Escherichia coli] [bcr] | 36.649 |
|  | two-component system response regulator BasR [Erwinia typographi] [pmrA] | 39.556 |
|  | cation acetate symporter [Wolinella succinogenes] [actP] | 53.728 |
|  | cation acetate symporter [Pseudomonas massiliensis] [actP] | 54.876 |
|  | MULTISPECIES: superoxide dismutase [Bordetella] [sodA] | 76.471 |
|  | MULTISPECIES: superoxide dismutase [Fe] [Ralstonia] [sodB] | 81.25 |
|  | MULTISPECIES: superoxide dismutase [Fe] [Ralstonia] [sodB] | 80.729 |
|  | superoxide dismutase [Fe] [Beggiatoa sp. 4572_84] [sodB] | 79.688 |
|  | superoxide dismutase [Cupriavidus pauculus] [sodB] | 79.167 |
